# Development of Very-Early-Onset Inflammatory Bowel Disease After Multiple Early-Life Antibiotic Exposures: A Case Report and Literature Review

**DOI:** 10.7759/cureus.33813

**Published:** 2023-01-16

**Authors:** Ángel A Miró-González, Sergio M Maldonado-Chaar, Raul Zambrana-Valenzuela, Ivonne M Iglesias-Escabi, Norma J Arciniegas-Medina

**Affiliations:** 1 Pediatrics, Ponce Health Sciences University, Ponce, PRI; 2 Pediatric Gastroenterology, Mayagüez Medical Center, Mayagüez, PRI; 3 Pediatrics, Mayagüez Medical Center, Mayagüez, PRI

**Keywords:** pediatric gastroenterology, gut dysbiosis and diseases, inflammatory bowel disease, fecal microbiota transplantation (fmt), very early onset inflammatory bowel disease

## Abstract

The use of antibiotics has increased drastically over the last few decades. Many antibiotics can target the commensal microbiota and promote gut dysbiosis. These alterations contribute to disease onset and exacerbation. Although the etiology of inflammatory bowel disease (IBD) is mostly unknown, it involves a complex interaction among host genetics, microbiota, environmental factors, and aberrant immune responses. Studies have shown a relationship between very-early-onset inflammatory bowel disease (VEO-IBD) and microbiota alterations. The case discussed in this report endorses the current clinical evidence for this interaction. This is an anonymous record review with no identifiers involving a 23-month-old female patient who was brought to the emergency department by her parents due to persistent bloody diarrhea. Eight days before the presentation, she had experienced watery diarrhea that progressed to bloody stools. The patient had a history of acute otitis media, acute enteritis, and right-arm cutaneous abscess, for which she had received multiple antibiotic therapies. Strategies to manipulate the microbiome through diet, probiotics, antibiotics, or fecal microbiota transplantation (FMT) may be used therapeutically to modulate disease activity. A high index of clinical suspicion for VEO-IBD should be maintained for patients with a history of multiple, recurrent antibiotic use. We believe this case report will raise awareness about the issue of early anaerobic antibiotic exposure and help prevent its unnecessary use and, consequently, prevent gut microbiota dysbiosis that can lead to VEO-IBD. Also, our literature review will hopefully prompt clinicians to consider alternative therapeutic options for this patient population, such as rebuilding intestinal microbiota composition to improve VEO-IBD activity.

## Introduction

Inflammatory bowel disease (IBD) is a multifactorial chronic inflammatory condition of the gastrointestinal tract characterized by the absence of a clear etiology [1,2,3]. It occurs due to the interactions between genetic and environmental factors leading to immunological responses and chronic inflammation in the intestine [3]. It is classified into two major phenotypes: Crohn's disease (CD) and ulcerative colitis (UC) [2,4]. IBD has evolved into a global disease with an increasing incidence in industrialized countries. It is estimated that 20-30% of new cases are diagnosed before the age of 20 years, most notably in younger children [2]. Very-early-onset inflammatory bowel disease (VEO-IBD) refers to a type of IBD diagnosed before the age of six years [5]. It is further subdivided into infantile IBD, diagnosed before age two, and neonatal IBD, diagnosed before 28 days of life [6]. VEO-IBD is characterized by increased disease severity, aggressive progression, strong family history of IBD, and poor response to conventional treatments [1,5]. VEO-IBD's behavior is often different in adolescents than in adults, as it is usually restricted to the colon and refractory to standard medical therapies [5]. While the etiology of IBD is not fully understood, it is currently hypothesized that IBD results from a complex interaction of genetics, immune dysregulation, and environmental triggers that exert their effects through alterations of the intestinal microbiota [7]. The term gut microbiota refers to the collection of all microorganisms that inhabit the gastrointestinal tract [8]. The human gut microbiota is a host-specific ecosystem that is inherited and matures in early childhood [8]. Recent evidence suggests that altered microbial communities, termed microbiota dysbiosis, and intestinal barrier impairment are associated with the development of chronic inflammatory disorders, including IBD [3]. A meta-analysis by Ungaro et al. showed that antibiotic use is strongly associated with IBD and that early use in life increases the risk of developing the condition [9]. Furthermore, it is widely recognized that antibiotics can alter the gut microbiota and promote dysbiosis, which has partly driven therapeutic options like fecal microbiota transplantation (FMT) [8,10,11,12].

We report the case of a VEO-IBD patient who was brought to our academic hospital due to bloody diarrhea episodes after receiving various antibiotic regimens during previous recurrent hospitalizations. This case report supports the current evidence on the possible pathogenesis of VEO-IBD as a consequence of microbiota dysbiosis. We hope that this case report and the literature review that accompanies it will aid in the early diagnosis of this condition and help physicians consider novel management modalities available for managing refractory and challenging cases. This is an anonymous record review with no identifiers.

## Case presentation

A 23-month-old female patient was brought to the emergency department due to several episodes of bloody diarrhea. The patient’s mother reported that the symptoms had been going on persistently for the past 40 days and had intensified in the last four days, prompting the mother to seek further medical attention. During these last four days, the patient experienced 26 episodes of bloody diarrhea associated with hypoactivity, weakness, weight loss, dry skin, and dry mouth. Furthermore, the mother denied any alleviating or aggravating factors, any history of recent travel, and any close contact with individuals with similar symptoms.

The patient's past medical history was remarkable for anemia refractory to iron supplementation, failure to thrive, and multi-antibiotic use due to recurrent hospitalizations for infections in the past two months, such as acute otitis media, forearm skin abscess formation, and acute enteritis. The antibiotic regimens during these hospitalizations included metronidazole for 10 days, amoxicillin-clavulanate for seven days, and vancomycin for seven days. Perinatal history revealed no complications during pregnancy. Feeding history showed lactation until six months of age, adequate response to formula transition, and a current irritant-free diet. Developmental history was significant for age-appropriate milestone achievements. Pertinent family history was significant for autoimmune disease in the father, specifically rheumatoid arthritis and uveitis, and autoimmune hypothyroidism in the paternal grandfather. Parents denied smoking, substance abuse, and alcohol consumption. The mother denied the use of any antibiotics during her pregnancy.

The patient’s vital signs were within normal limits at the time of evaluation. On physical examination, the patient was pale and had dry mucous membranes. Abdominal examination showed bowel sounds present in all quadrants; the abdomen was depressible and soft with voluntary guarding. Bright red blood per rectum was observed on rectal examination. The remainder of the physical examination was unremarkable. Complete blood count was notable for leukocytosis with a normal differential, hemoglobin that trended from 11.1 g/dL on admission to 8.1 g/dL being the lowest value reported, and thrombocytosis. The comprehensive metabolic panel was without major electrolytic disturbances, with stable renal function, normal liver enzymes, and hypoalbuminemia. Inflammatory markers such as erythrocyte sedimentation rate (ESR) and C-reactive protein (CRP) were elevated. Iron studies revealed elevated ferritin levels. Clostridium difficile toxin and antigen studies were negative. The stool ova and parasites exam was negative. Stool cultures were negative for viruses and pathogenic bacteria. Overall, there was no evidence of microbial infection. Stool workup was also remarkable for elevated white and red blood cells with elevated stool calprotectin and lactoferrin levels. Additionally, the immunology service was consulted and immunological testing was performed, including Dihydrorhodamine flow cytometry, lymphocytic analyses, and immunoglobulin levels. These studies revealed no immune deficiencies but showed an elevated lymphocytic count, and an increased absolute neutrophil count. Vaccine titers revealed a deficiency only in the diphtheria component due to the patient missing vaccine doses due to parental vaccine aversion preferences.

**Figure 1:**
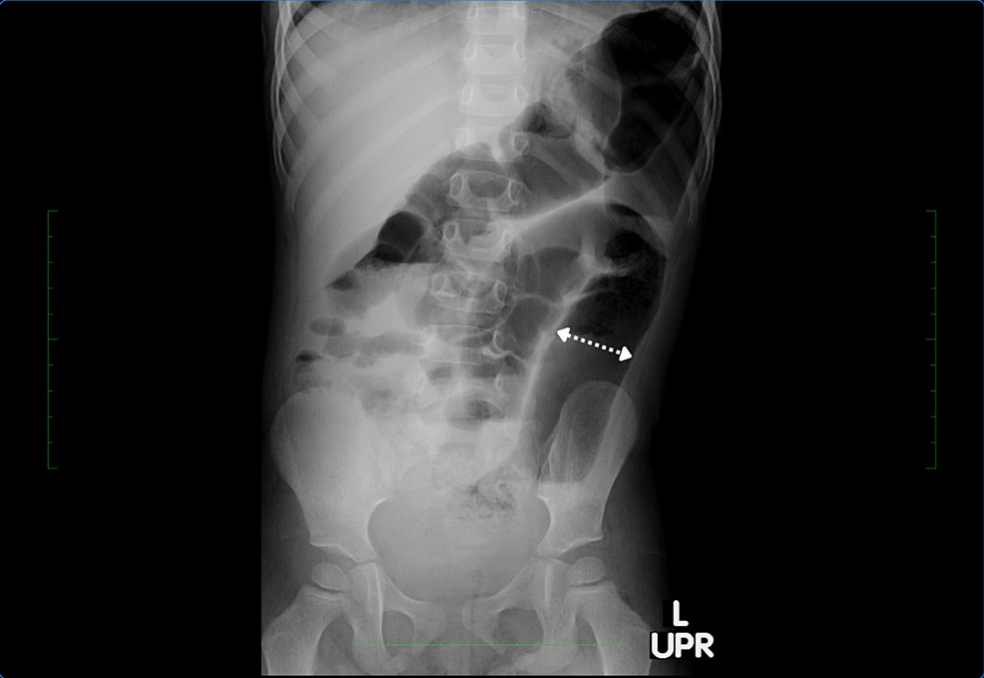
Abdominal radiography The figure demonstrates abdominal radiography showing air-filled bowel distention (indicated by the arrow) without pneumatosis

**Figure 2:**
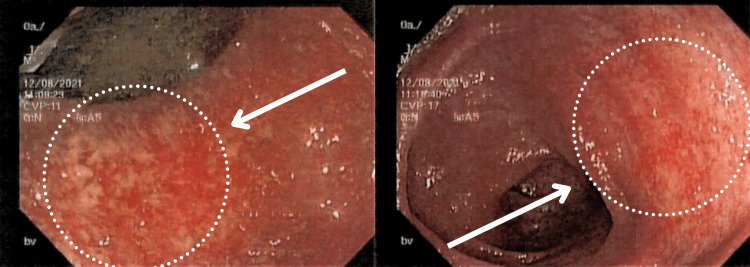
Colonoscopy view of the rectosigmoid junction The figure demonstrates the colonoscopy view of the rectosigmoid junction with exudated, friable, erythematous mucosa (enclosed within circles pointed by arrows) and superficial erosions with a decreased vascular pattern

**Figure 3:**
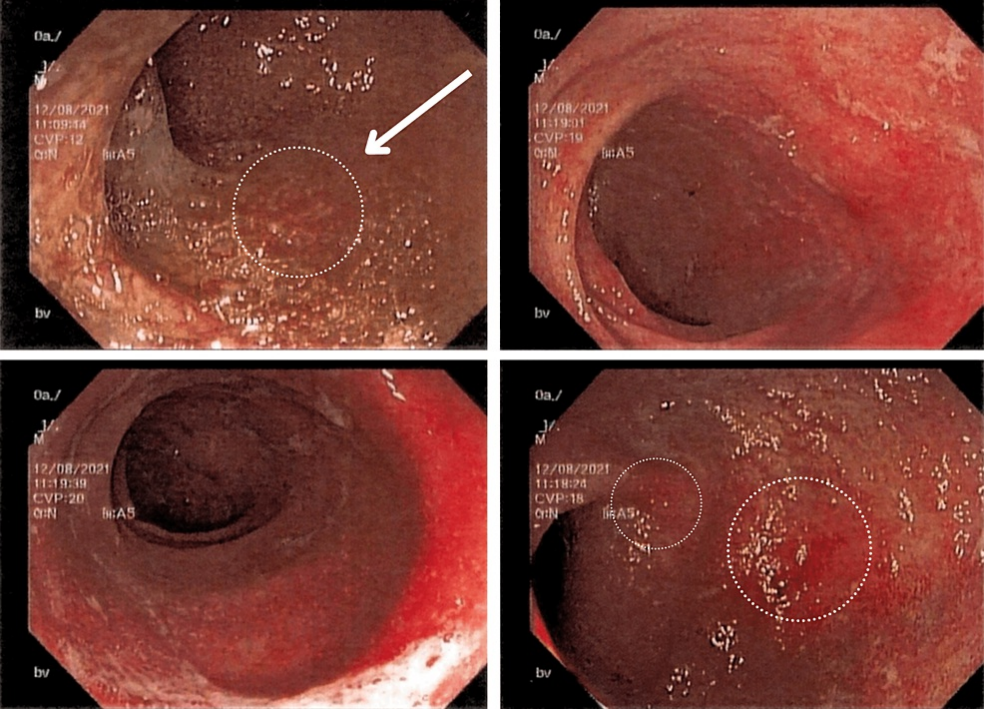
Colonoscopy view of the descending colon The figure demonstrates the colonoscopy view of the descending colon with erythematous and edematous mucosa (enclosed within circles and evident in the right-upper and bottom-left images)

**Figure 4:**
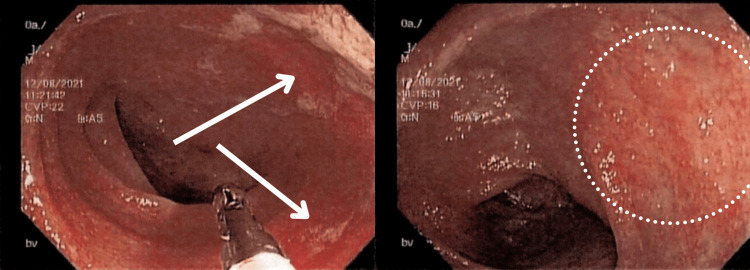
Colonoscopy view of the transverse colon This figure demonstrates the colonoscopy view of the transverse colon with erythematous and edematous mucosa (arrows in the left image and circle in the right image)

The patient’s refractory rectal bleeding without a clear etiology prompted further imaging studies and direct visualization procedures. Abdominal ultrasound was negative for any acute abdominal process; abdominal radiography showed air-filled bowel distention without pneumatosis (Figure 1), and Meckel's scan revealed no evidence of ectopic gastric mucosa. Given the patient’s clinical picture, family history of autoimmune disorders, and history of multiple antibiotic use in the early months of her life, it was proposed that the patient was undergoing an inflammatory rather than an infectious process. Subsequently, a colonoscopy and esophagogastroduodenoscopy (EGD) with biopsies were performed to rule out IBD. The colonoscopy with biopsy revealed an exudated, friable, erythematous mucosa at the rectosigmoid junction with superficial erosions and decreased vascular pattern (Figure 2) and erythematous and edematous mucosa at the transverse and descending colon (Figures 3, 4).

**Figure 5:**
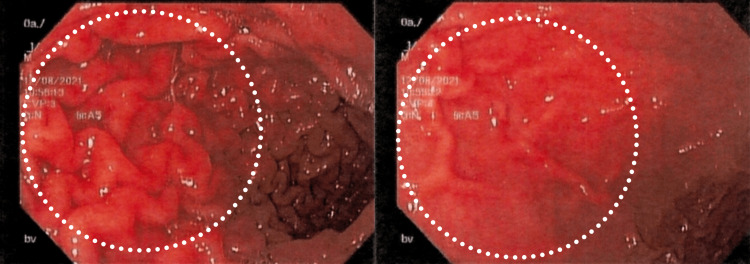
Endoscopic view of the gastric body The figure demonstrates the endoscopic view of the gastric body with a linear erythematous mucosa (enclosed within circles)

**Figure 6:**
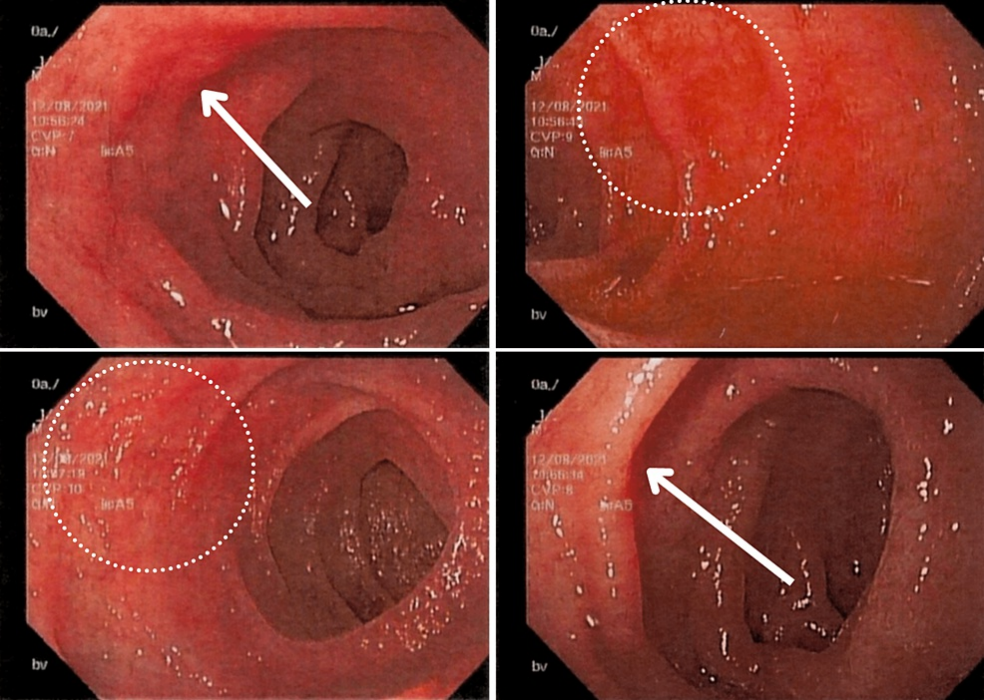
Endoscopic view of the first duodenal segment The figure demonstrates the endoscopic view of the first duodenal segment showing swollen mucosa (arrows in the top-left and bottom-right images, circles in the top-right and bottom-left images)

**Figure 7:**
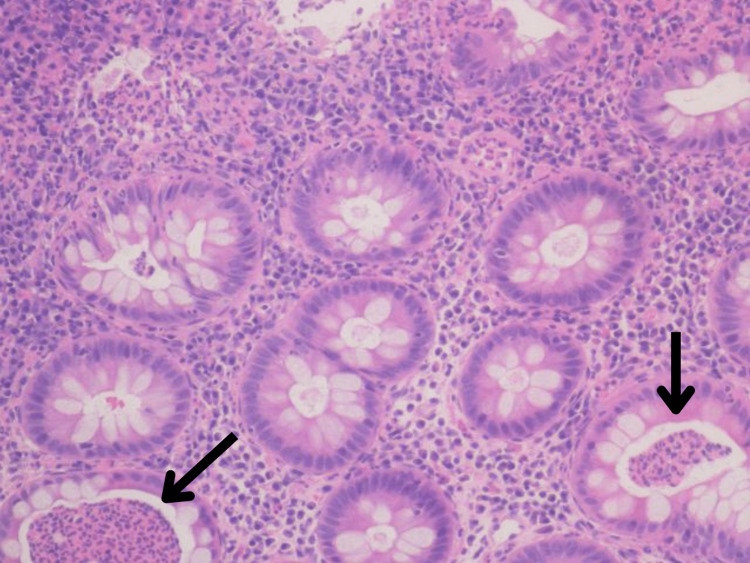
Histopathology sample of the sigmoid colon The figure shows the histopathologic sample of the sigmoid colon with evident mild acute colitis and acute neutrophilic crypt abscesses (indicated by arrows)

The EGD revealed linear erythematous mucosa in the gastric body and the first portion of the duodenum with swollen mucosa (Figures 5, 6). The microscopic findings of both studies revealed mild acute-on-chronic gastritis with crypt abscesses, mild acute-on-chronic duodenitis with mild villous blunting, mild acute-on-chronic colitis in the transverse and descending colon, and mild acute colitis with acute neutrophilic crypt abscesses in the sigmoid and rectum (Figure 7).

Considering all the clinical, laboratory, imaging, endoscopic, and histologic findings mentioned above, a diagnosis of VEO-IBD, most likely a UC phenotype with the involvement of the upper gastrointestinal tract, was confirmed. Since the possibility of a genetic cause had to be excluded, an Invitae Primary Immunodeficiency Panel and Invitae Monogenic Inflammatory Bowel Disease Panel (Invitae Corp., San Francisco, CA) were ordered. Out of the 429 genes tested, only one variant of unknown significance associated with VEO-IBD was detected: ANKZF1 Partial Deletion of Exon 14. However, according to the report, the available evidence of this variant is currently insufficient to determine its role in the development of VEO-IBD; thus, the functional significance of this variant is currently unknown. Therefore, assigning a genetic cause for the patient's condition was deferred. Nonetheless, we believe that our patient has a genetic predisposition to developing VEO-IBD that was precipitated by early-life antibiotic use since VEO-IBD is a multifactorial disease that is rooted in the interaction between environmental and genetic components in the gastrointestinal tract microbiome. Considering this as well as the early-life exposure to multiple antibiotics during recent recurrent hospitalizations, the diagnosis of VEO-IBD as a consequence of microbiota dysbiosis was established. The patient was started on corticosteroid therapy and mesalamine, which led to a significant improvement. Once the patient was stabilized, bloody diarrhea episodes decreased appropriately, inflammatory markers such as CRP and ESR decreased, and fecal calprotectin levels decreased, she was discharged on sulfasalazine with folic acid and corticosteroid maintenance therapy. In the six months following her discharge, the patient has had multiple relapses with persistently elevated inflammatory markers and deterioration of her condition (despite an immunomodulator induction trial with infliximab) for which alternative therapeutic options are being considered.

## Discussion

Antibiotics use has been on the rise over the past decade globally, with increased use in medicine to prevent and treat pathogen-related diseases [13]. As a result, some geographic regions have witnessed an increasing trend in the incidence of VEO-IBD, amounting to around 7.2% per year [14]. Studies have shown that the use of antibiotics precipitates an inflammatory response in mammals, likely due to the decrease in microbiota diversity and commensal gut bacteria translocation [15]. In fact, a study by Anthony et al. found that after a five-day treatment with antibiotics, the reduction of the microbiota population and its diversity was first observed on day six itself, the very next day after antibiotic completion [16]. Also, the genetic influence of IBD has remained stagnant, leading to the premise that an environmental modifier, such as a change in microbiota or an immune response, drives the increase in incidence in individuals of younger age groups [2]. Hence, this is a crucial group that merits more investigation in terms of researching the cause of IBD further and establishing a relationship. This relationship is explored extensively in our current literature review, which suggests a strong association between antibiotic usage in early life and VEO-IBD development. This can be seen in our case report, where our patient was exposed to multiple antibiotics early in life, including vancomycin, metronidazole, and amoxicillin-clavulanate. Additionally, our patient was exposed to these in conditions, such as otitis media and acute enteritis, where the antibiotics could have been held until a deterioration of her condition might have indicated their use.

IBD is a gastrointestinal condition resulting from an abnormal immune response to environmental exposures, such as microbiota dysbiosis, in a genetically predisposed individual [17]. It is a multifactorial disease that originates from the interplay of both environmental and genetic factors in the intestinal microbiome [18]. Even though the exact pathophysiological mechanism in VEO-IBD is still a mystery, it is thought to stem from bacterial interactions with the host [19]. The interactions were discovered to be cyclic, showing an increase in disease-causing bacterial strains with a corresponding increase in the human immune response during active intestinal inflammation [20]. According to research into the mucosal immune response, patients with UC mount an immunoglobulin response against endogenous bacterial components [20]. A lack of specific bacteria, such as anaerobes, can contribute to VEO-IBD along with the immunoglobulin response to bacterial compounds [20]. Anaerobic bacteria of the colon produce short-chain fatty acids, which are the principal energy source for intestinal epithelial cells and are essential for barrier integrity and immune-activation inhibition [21]. Depletion of short-chain fatty acid-producing organisms may impair already-weak intestinal epithelial cells, allowing commensal or low-pathogenic bacteria to invade and activate immunocompetent cells [20]. This could explain the clinical evidence reported by Kronman et al. in a retrospective cohort study, where exposure to antianaerobic antibiotics was associated with IBD development in children [22].

Going into greater detail, a study by Anthony et al. found that a single antibiotic exposure can cause a reduced abundance of bacteria from Eubacterium genera, an important gut commensal, but increased abundance from the genus Erysipelotrichaceae [16]. Erysipelotrichaceae species are known to be highly immunogenic and have been linked to IBD with an increase in its abundance post-antibiotic use. Further support for the role of the microbiome in IBD has been found in studies based on genetically engineered mouse models of IBD, which have shown that for intestinal inflammation to occur the commensal enteric bacteria are needed, and in their absence, such as in a germ-free state, this animal models do not progress to an inflammatory state [23]. Of the known IBD susceptibility genes, the majority are associated with host mucosal barrier function, which further supports our point [24]. In our case, we believe that the genetic component that predisposed our patient to develop IBD could be attributed to the patient's family history of autoimmune disease on the father's side, keeping in mind that IBD, being a multifactorial condition, has an autoimmune factor. We also believe that the environmental component that interacted with the patient's mentioned predisposition was the exposure to multiple antibiotics that are widely known to target the anaerobic bacterial population, namely metronidazole and amoxicillin-clavulanate, via the mechanism explained above. This interplay between early-life exposure to these types of antibiotics and the patient's genetic predisposition could be the pathogenesis of our patient's VEO-IBD.

VEO-IBD patients commonly present with a broad variety of clinical manifestations, which could be classified into gastrointestinal and extraintestinal symptoms [25]. Gastrointestinal symptomatology involves bloody or mucus-containing diarrhea, emesis, failure to thrive, perianal skin tags, or fistulas [17,25]. Extraintestinal symptoms include uveitis, arthralgias, arthritis, intermittent fevers, folliculitis, and dermatologic manifestations such as erythema nodosum and pyoderma gangrenosum [25]. Nonetheless, of the mentioned features, the ones that should increase the index of clinical suspicion for VEO-IBD are intermittent fevers, failure to thrive, severe perianal disease, frequent infections, and weight loss [22]. It is also essential to consider the patient’s family history to assess any predispositions to developing VEO-IBD. A family history of consanguinity, autoimmune conditions, inflammatory diseases, hemophagocytic lymphohistiocytosis, arthritis, susceptibility to infections, and malignancy should be noted and raise suspicion for VEO-IBD [17]. Our patient presented with multiple episodes of bloody diarrhea associated with failure to thrive, weight loss, and a history of frequent infections, which are all features that should increase the index of clinical suspicion as mentioned. Even though these features raised suspicion for VEO-IBD, other common infectious and inflammatory etiologies that could have precipitated these symptoms had to be ruled out.

The patient’s family history of autoimmune disease raised the suspicion that she was genetically predisposed to developing VEO-IBD. Also, current literature has demonstrated that antibiotic use in infancy changes the intestinal microbiome and may exacerbate IBD in susceptible individuals [2]. This further raised the clinical suspicion for VEO-IBD since our patient had a history of repeated antibiotic usage. Furthermore, although it may be challenging to suspect VEO-IBD, it is crucial to keep this diagnosis on the list of differentials to avoid delayed treatment [25]. The VEO-IBD in our patient was further characterized as UC-type based on the histologic findings that are characteristic of this condition, most notably the evidence of acute colitis and acute neutrophilic crypt abscesses. Additionally, our patient's condition was complicated by upper gastrointestinal tract involvement as evidenced by endoscopic findings. UC with upper gastrointestinal tract lesions is a rare finding [26]. The concept of gastroduodenal ulcerative colitis (GDUC) was presented by Hori et al. and, although the etiology of this condition remains unclear, studies have demonstrated that it may be associated with the disproportion of immune responses in a genetically predisposed individual to bacterial antigens, resulting in a disproportionate autoimmune response to the gastroduodenal epithelium [26]. All of the above-mentioned findings explain our patient's UC with upper gastrointestinal involvement, and it is important to note that upper gastrointestinal involvement does not necessarily indicate that the patient automatically has CD. It is also imperative to take into account the endoscopic and histologic evidence in order to arrive at the correct diagnosis.

The diagnostic approach to VEO-IBD primarily aims to identify patients who will benefit from nonstandard therapies or those who are at risk of complications that require close observation [17]. A standard comprehensive laboratory evaluation should include a complete blood count, comprehensive metabolic profile, and inflammatory markers [17]. Also, stool lactoferrin and calprotectin could be used since both may be elevated during infectious, inflammatory, and allergic gastrointestinal disorders [25]. In patients presenting with chronic diarrhea, infection by pathogens, depending on the patient’s risk profile, should be ruled out with a stool culture regardless of age [25]. For patients older than 12 months, testing for *Clostridioides difficile as *a possible etiology should be considered, especially in the setting of previous antibiotic use [25]. Once other etiologies have been ruled out and if the lab tests, clinical manifestations, and physical exam findings support the diagnosis, it is recommended to proceed with radiological imaging and direct visualization procedures [25]. Radiological imaging such as abdominal ultrasound, magnetic resonance enterography (MRE), and CT scan are widely employed at most institutions as part of the management [17]. However, since MRE poses difficulties in young children and CT involves extensive radiation, an ultrasound can be used to delineate the extent of the disease [17]. Furthermore, endoscopic evaluation helps establish if the findings are compatible with an infectious, inflammatory, or allergic process [25]. Also, a histopathologic examination substantially aids in the diagnosis of VEO-IBD. The common findings include changes in tissue architecture, noncaseating granulomas, lamina propria with increased leukocyte content, and crypt abscesses [25].

If initial screening studies (laboratory, endoscopic, and radiologic studies) to rule out other causes of presenting symptoms raise concern for VEO-IBD, additional second-tier testing should be considered [17]. Current recommendations propose that the clinical presentation should guide genetic testing and the index of clinical suspicion, based on the availability of resources, institutional policies, and costs [27]. Immunology consultation for all VEO-IBD patients is indicated to investigate an underlying immunodeficiency [25]. These include the evaluation of humoral immunity and antibody deficiency. Thus, a patient with suspected VEO-IBD may benefit from immunoglobulin and vaccine titers to screen for memory deficits [17]. Testing should also include a neutrophil respiratory burst assay to evaluate chronic granulomatous disease [17]. Targeted genetic panels or next-generation sequencing are clinically indicated if no monogenetic etiology is suspected or if directed testing does not reveal any specific findings. When specific variants are identified, this poses tremendous clinical and therapeutic implications [25]. If genetic testing is unrevealing, this does not necessarily imply that the disease process does not involve a genetic component. This is because the current list of known genes associated with VEO-IBD is insufficient [25]. The decision to proceed with genetic testing is best made in coordination with a multidisciplinary team approach that includes genetic counseling availability and support. This diagnostic approach, including all of the above-mentioned studies, was utilized in our patient's workup, where the diagnosis of IBD was confirmed, and other etiologies, including immunogenic causes, were excluded. Thus, we propose that early exposure to antibiotics caused VEO-IBD in our patient, a predisposed individual.

It has been described in the medical literature that VEO-IBD patients manifest a highly severe condition that is resistant to conventional treatment options [2]. These patients have a higher likelihood of receiving treatment with steroids, immunomodulators, and surgery in the first year post-diagnosis when compared to adults with IBD [28,29]. Many cases of VEO-IBD require management that includes parenteral nutrition, immunosuppression, hematopoietic stem cell transplantation, and surgery [30]. Our patient was treated with steroids and immunomodulators to induce remission; nonetheless, after initial improvement, she has suffered multiple relapses and has deteriorated clinically with corresponding persistently elevated inflammatory markers in the sixth-month post-discharge period, for which other therapeutic alternatives are being explored.

FMT has been gaining significant attention since a recent pilot randomized control trial of FMT for UC in young children showed promising results for FMT as a medical treatment of VEO-IBD [12]. FMT involves the transferring of a fecal matter solution from a donor's to a recipient's intestine to alter the gut microbiota and grant a clinical benefit [31]. In a case report by Yodoshi and Hurt, a five-year-old female was treated using FMT for her diagnosed UC that had affected the entire colon at the age of two years and 10 months [11]. The patient achieved clinical remission after one week, with a pediatric ulcerative colitis activity index (PUCAI) score of 0. After three months, the fecal analysis demonstrated that the patient's intestinal flora was similar to the donor's fecal matter. As a result, clinical remission of UC activity was sustained for a period of two years since the study was published [11]. An adequate understanding of the mechanisms of VEO-IBD and disease advancement is needed to devise targeted therapeutic strategies for this patient population and improve their outcomes [17]. There has been only one pilot randomized control trial report of FMT for UC in young children, in which 25 patients with UC were randomized to twice-weekly enema therapy or placebo for six weeks. At six weeks, biochemical markers such as CRP and fecal calprotectin showed improvement with trends towards improved clinical response [12]. We recommend that researchers and clinicians conduct more extensive studies exploring FMT as a possible treatment option in VEO-IBD patients who do not respond to conventional therapies. This could provide a possible treatment option for a condition that is highly resistant to current treatments.

## Conclusions

Based on our literature review, antibiotic exposure at an early age can promote gut dysbiosis within the first week. These alterations can contribute to disease onset and exacerbation pertaining to VEO-IBD. Finding the mechanisms by which specific antibiotics affect the microbiome may prove to be worthy information for developing therapeutic interventions in terms of modulating the intestinal microbiota in cases refractory to conventional treatment. Furthermore, it may be proper to limit early antibiotic exposure unless it is absolutely necessary, especially in patients at an increased risk for VEO-IBD. In addition, a high index of clinical suspicion for VEO-IBD should be maintained for patients with a history of early exposure to anaerobic antibiotics, and previous antibiotic exposures should be thoroughly assessed. Finally, We hope this report will contribute to raising awareness about the issue of early anaerobic antibiotic exposure in order to prevent unnecessary use and thereby prevent gut microbiota dysbiosis that can lead to VEO-IBD.
